# Distinct pathways for the absorption and metabolism of β-carotene and zeaxanthin in the mouse intestine

**DOI:** 10.1016/j.jlr.2025.100758

**Published:** 2025-02-17

**Authors:** Sepalika Bandara, Aicha Saadane, Tong Shen, Daryna Yakovleva, Rakhee Banerjee, Yanqi Zhang, J. Mark Brown, Johannes von Lintig

**Affiliations:** 1 Department of Pharmacology, School of Medicine, Case Western Reserve University, Cleveland, OH, USA; 2 Department of Cancer Biology, Lerner Research Institute of the Cleveland Clinic, Cleveland, OH, USA

**Keywords:** Carotenoid cleavage oxygenases, carotenoids, intestine, lipid transfer proteins, retinoids, transport

## Abstract

Carotenoids, essential nutrients for eye health, are absorbed in the intestine to support vitamin A homeostasis and provide cellular protection. This process involves the lipid transporters scavenger receptor class B type 1 (SR-B1, encoded by *Scarb1* gene) and Niemann-Pick C1-Like 1 (NPC1L1), which load these dietary lipids into the plasma membrane of intestinal enterocytes. However, the precise contribution of these transporters to carotenoid absorption, the putative involvement of Aster proteins in their downstream movement, and the interactions with their metabolizing enzymes, β-carotene oxygenase 1 (BCO1) and β-carotene oxygenase 2 (BCO2), remain incompletely understood. Here, we investigated carotenoid metabolism in the mouse intestine using pharmacological and genetic approaches. We observed that ezetimibe, an NPC1L1 inhibitor, reduced zeaxanthin but did not affect β-carotene absorption. Aster-C, highly expressed in enterocytes, bound zeaxanthin in biochemical assays. In mice, Aster-C deficiency led to upregulation of *Gramd1b* (Aster-B) expression and increased zeaxanthin bioavailability. We further showed that BCO1 directly interacted with membranes to extract β-carotene for retinoid production, indicating that vitamin A production is Aster protein-independent. This observation is consistent with the finding that the intestine-specific transcription factor ISX, the master regulator of vitamin A production, controlled *Scarb1* and *Bco1* expression but had no effect on *Gramd1a*, *b*, or *c*, encoding Aster proteins in intestinal enterocytes. Together, our study revealed distinct pathways for β-carotene and zeaxanthin absorption and metabolism, offering new insights into carotenoid bioavailability and potential strategies to optimize dietary carotenoid intake for improved eye health.

Carotenoids, yellow to red dietary pigments, play crucial roles in human physiology, acting as antioxidants and blue light filters in the retina (1, 2, 3). The pigments are also essential precursors of vitamin A, necessary for synthesizing retinaldehyde, the chromophore of visual pigments, and all-*trans*-retinoic acid, a hormone-like metabolite regulating gene expression via nuclear receptors in the eyes and other organs (4, 5). Inadequate carotenoid supplies have been associated with vitamin A deficiency syndrome as well as chronic ocular diseases such the age-related macular degeneration (6, 7, 8, 9), the leading cause of blindness in elderly persons in industrialized countries.

Dietary carotenoids are acquired by intestinal enterocytes from mixed micelles and travel in lipoproteins throughout the body. Clinical studies have unveiled that genetic polymorphisms in the genes encoding scavenger receptor class B type 1 (SR-B1, encoded by the *Scarb1* gene) and Niemann-Pick C1-Like 1 protein (NPC1L1) are associated with serum levels of carotenoids (10, 11, 12). These proteins facilitate the absorption of dietary cholesterol as well as tocopherols, phylloquinones, and carotenoids (13, 14, 15).

In enterocytes, carotenoids can follow two primary metabolic fates (14). Provitamin A carotenoids like β-carotene are enzymatically cleaved by β-carotene-oxygenase-1 (BCO1) into retinaldehyde, which is then reduced to retinol (ROL) and subsequently esterified to retinyl esters (REs) (16). REs, along with other dietary lipids, are packaged into chylomicrons for transport (17). Non-provitamin A carotenoids, such as lutein and zeaxanthin, are either incorporated into chylomicrons or directed to mitochondria, where β-carotene-oxygenase-2 (BCO2) converts them into apocarotenoids (18, 19). However, our understanding of how carotenoids move in cells and interact with BCO1 and BCO2 for metabolic processing remains incomplete.

Previously, Aster proteins have been identified to operate downstream of SR-B1 and NPC1L1, in cholesterol uptake. These proteins aid in the transportation of cholesterol from the plasma membrane (PM) to other cellular membranes through non-vesicular transport (20). Aster proteins exhibit a tripartite structure featuring a StART-like lipid binding domain flanked by an N-terminal GRAM domain, and a C-terminal membrane anchor domain (21). This structural arrangement enables Aster proteins to establish contact points between the endoplasmic reticulum (ER) and PM, as well as between the ER and mitochondria (22). Studies in mice revealed that Aster proteins play crucial roles in cholesterol uptake in enterocytes, steroidogenesis, and reverse cholesterol transport (21, 23, 24).

We recently showed that StART-like domain of Aster-A and B can also bind carotenoids such as zeaxanthin, and lutein (25). Furthermore, our investigations in human cell lines have revealed that Aster-B efficiently transports these carotenoids from PM to mitochondria (26). We here applied genetic, biochemical, and pharmacological approaches to study the metabolism of β-carotene and zeaxanthin in the mouse intestine. Particularly, we determined the contributions of SR-B1 and NPC1L1 to the absorption of the two different carotenoids and studied whether Aster proteins facilitate their downstream movement and interaction with BCO1 and BCO2.

## Materials and methods

### Materials

All chemicals, unless otherwise specified, were purchased from Fisher and Sigma-Aldrich. Carotenoids were a gift from Dr Adrian Wyss (DSM). DNA isolation kits were purchased from Qiagen.

### Plasmid construction and site-directed mutagenesis

The murine Aster-C lipid binding domain construct was designed using *Gramd1c* transcript variant 2 (NM_001172107.1) from amino acids 100 to 312. The open reading frame (ORF) was amplified from mouse liver cDNA and cloned into pMAL C5x vector using SbfI and XmnI restriction sites. The sequence was confirmed by Sanger sequencing. The cloning of human BCO1 cDNA has been previously described (27). We here cloned the cDNA into the pMAL C5x vector using SbfI and XmnI restriction sites. We used the Quick change site directed mutagenesis kit (Agilent) to generate C102A, I105E, and L113D mutant variants of human BCO1 using the following primers C102A forward primer 5′-CTTTGGAAAATATGTTTTTGGCGGGGTCCGGATAGGCCATT-3′; reverse primer 5′-AATGGCCTATCCGGACCCCGCCAAAAACATATTTTCCAAAG-3′; I105E forward primer 5′-AGTAGGAGAAAGCTTTGGAAAACTCGTTTTTGCAGGGGTCCGGATAG-3′; reverse primer 5′-CTATCCGGACCCCTGCAAAAACGAGTTTTCCAAAGCTTTCTCCTACT-3′; L113D forward primer 5′-GTGAAATCGGGGATGGTGTGAGAATCGTAGGAGAAAGCTTTGGAAAATA-3′; reverse primer 5′-TATTTTCCAAAGCTTTCTCCTACGATTCTCACACCATCCCCGATTTCAC-3′. The C102A/L113D double mutation was generated by using the C102A hBCO1 plasmid and the L113D primers.

### Protein expression and purification

Aster-C protein was expressed as maltose binding protein (MBP) fusion protein. Protein expression and purification were carried out as in our previous work (25). Briefly, Aster-C pMAL plasmid was transformed either into XL1-Blue *E. coli* cells carrying genes for carotenoid biosyhthesis for the production of carotenoprotein complexes or into BL21 *E. coli* cells for apo-protein production. Then, 500 ml LB broth was inoculated with the respective pre-culture and grown at 37°C until it reached a OD600 of 0.5. Then, protein expression was induced with 0.2 mM IPTG and the culture was grown overnight in the dark at 30°C. The cells were harvested by centrifugation and stored at −80°C until further use. Apoprotein and carotenoprotein complexes were purified using affinity chromatography with amylose resin.

### Cell-based BCO1 enzymatic activity

XL1-Blue strain of *E. coli*, engineered to produce β-carotene (28), was transformed with the different human BCO1 expression plasmids. Bacterial cells were grown in an LB medium at 37°C. Once an optical density of one absorbance unit at 600 nm (one OD_600_) was achieved, bacterial cell cultures were transferred to room temperature, and expression of BCO1 variants was induced by isopropyl-D-1-thiogalactopyranoside at a final concentration of 0.2 mM. Ferrous sulfate at the concentration of 25 mg/L was added at the time of induction. Expression continued at 16°C for 16 h. Cells were harvested by centrifugation at 4000 *g* for 15 min at 4°C. Carotenoid extraction from the bacterial pellet was performed under dim red light (<600 nm). A bacteria pellet (20 OD_600_) was dissolved in 200 μl 2 M hydroxylamine (pH 6.8) and 200 μl methanol. Then 400 μl acetone and 500 μl hexane was used to extract carotenoids and retinoids. Phase separation was achieved by centrifugation at 4000 *g* for 30 s. The organic phase was collected and the extraction was repeated. The combined organic phases were collected and dried in a Speedvac (Eppendorf). Lipids were dissolved in 150 μl hexane: ethylacetate (90:10 v/v) and subjected to HPLC analysis.

### Cell-free BCO1 enzyme assay

β-Carotene (2000 pmol) was dissolved in acetone and mixed with 3% w/v DMN in 100% ethanol. The solvent was evaporated with dry vacuum centrifugation (Eppendorf). The carotenoid detergent mixture was dissolved with 50 μl of reaction buffer (20 mM Tricine, 150 mM NaCl, 0.5 mM TCEP at pH 7.4). Fifty microlitre of enzyme solution (50 μg) was added and the mixture was incubated at 37°C under shaking at 600 rpm in a thermomixer (Eppendorf) for 15 min. The reactions were stopped by the addition of 100 μl 2 M hydroxylamine (pH 6.8), 200 μl methanol. After 10 min, 400 μl of acetone and 500 μl of hexane were added. Organic and aqueous layers were separated with a 10 s spin at 3000 *g*. The organic layer was collected and evaporated in a dry vacuum centrifuge (Eppendorf) and dissolved in hexanes: ethyl acetate, 90:10 (v/v) and subjected to HPLC analysis.

### UV-visible spectra of carotenoprotein complexes and HPLC analysis

The absorption spectra of the carotenoprotein complexes were recorded using a spectrophotometer (Cary 60, Varian). Hundred microgram of the carotenoprotein was used to extract carotenoids for HPLC analysis. Hundred microlitre methanol, 400 μl acetone, 300 μl diethyl ether and 200 μl petroleum ether were used to extract carotenoids. The organic layer was collected and vacuum dried. The residue was reconstituted in 70:30 hexanes/ethyl acetate and analyzed with normal phase HPLC. HPLC analysis was carried out with an Agilent 1260 Infinity Quaternary HPLC system equipped with a pump (G1312C) with an integrated degasser (G1322A), a thermostat column compartment (G1316A), an autosampler (G1329B), a diode-array detector (G1315D), and online analysis software (Chemstation). The analyses were carried out at 25°C using a normal phase Zorbax Sil (5 μm, 4.6 × 150 mm) column (Agilent Technologies). Chromatographic separation was achieved by isocratic flow at 1.4 ml per minute.

### Mouse strains and husbandry

The generation *Gramd1c*^*−/−*^ (29), *Bco1*^*−/−*^ (16), *Bco2*^*−/−*^ (18), and *Isx*^*−/−*^ (30) mice has been previously described. Mice were held in vivariums at Cleveland Clinic Foundation and Case Western Reserve University. All mouse experiments in these studies were conducted using approved protocol by Case Western Reserve University’s Institutional Animal Care and Use Committee (IACUC) and adhered to the guidelines of the Association for Research in Vision and Ophthalmology Statement for the Use of Animals in Ophthalmic and Vision Research. All mice were bred on a standard chow diet (Prolab RMH 3000, LabDiet).

Prior to the dietary intervention, mice were fed on AIN-93G Growing Rodent Diet without vitamin A supplement (VAD) for one week as a washout phase. The same diets containing β-carotene and 3R, 3′R-zeaxanthin were prepared by Research Diets (New Brunswick) by incorporating a water-soluble formulation of β-carotene and 3R, 3′R-zeaxanthin (DSM). Ezetimibe was purchased from Sigma and dissolved in ethanol. The ezetimibe solution was mixed in a blender with β-carotene and zeaxanthin diets to achieve a final concentration of 100 mg/kg diet. This corresponded to a dose of approximately 16 mg/kg × day as used in previous mouse studies (31). Then, pellets were formed and air-dried. Mice were fed with these diets for the indicated time periods. At the end of the dietary intervention, mice were sacrificed, tissues were immediately dissected, weighed, and snap-frozen in liquid nitrogen, and stored at −80°C until further analysis.

### Carotenoid extraction from mice tissues and HPLC analysis

Ten to twenty milligram of tissue was taken for carotenoid extraction. β-Carotene was extracted with 100 μl PBS, 100 μl methanol, 400 μl acetone and 500 μl hexanes. The organic layer was separated, vacuum dried, and reconstituted in hexanes/ethyl acetate (90:10 v/v) and analyzed using isocratic elution of 90:10 hexanes/ethyl acetate with normal phase HPLC as described above. Zeaxanthin was extracted with 100 μl PBS, 100 μl methanol, 400 μl acetone, 300 μl diethyl-ether and 200 μl petroleum ether. The organic layer was separated vacuum dried and reconstituted in hexanes/ethyl acetate (70:30 v/v) and analyzed using isocratic elution of 70:30 hexanes/ethyl acetate with normal phase HPLC using the above mentioned HPLC system.

### qRT PCR gene expression analysis

Approximately, 10 mg of tissue was used to isolate RNA using the Trizol method. RNA concentration and purity were determined with a Nano-drop spectrophotometer. cDNA was generated using High capacity RNA to cDNA kit (Applied Biosystems). Gene expression analysis was carried out by real-time quantitative PCR using an Applied Biosystems Real-time PCR instrument with Taq Man probes (Applied Biosystems). Primers used for analysis were *Actinb* (Mm02619580_g1), *Bco1* (Mm01251350_m1), *Bco2* (Mm00460051_m1), *Isx*, *Npc1l1* (Mm01191972_m1), *Gramd1a* (Mm00472081_m1), *Gramd1b* (Mm00555274_m1), *Gramd1c* (Mm00463408_m1), and *Scarb1* (Mm450234_m1). Amplification was carried out using TaqMan polymerase Fast universal PCR Master MIX (2 ×) No Amp Erase, UNG (Applied Biosystems) following the manufacturer’s protocol. Twenty nanograms cDNA was used per 10 μl reaction. Gene expression levels were normalized to the expression of housekeeping gene *Actinb* using the ΔΔCt method.

### Statistical analysis

Data shown are the mean ± standard deviation (SD). Analysis was performed using unpaired two tail *t* test and one-way ANOVA using Graph pad Prism 10.2 software, and results were considered significant at ∗*P* < 0.05, ∗∗*P* < 0.005, ∗∗∗*P* < 0.0001.

## Results

### Ezetimibe treatment decreases xanthophyll but not β-carotene absorption in mice

**Fig. 1:**
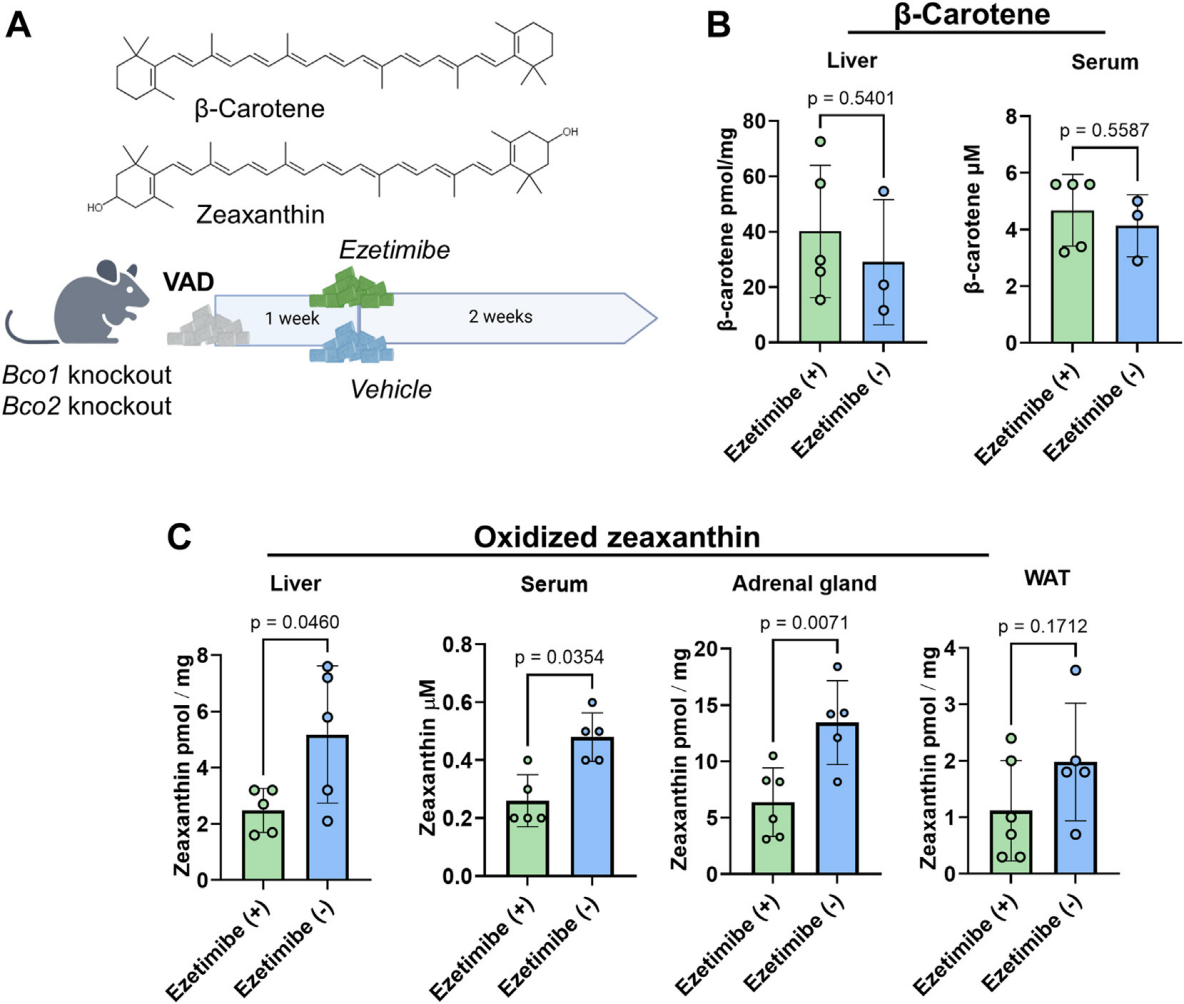
Effects of ezetimibe treatment on zeaxanthin and β-carotene metabolism in mice. A: Chemical structures of β-carotene and zeaxanthin and scheme of the feeding study. Mice were subjected to one-week feeding with vitamin A and a carotenoid-free diet. Then, they were subjected to diets containing β-carotene (25 mg/kg) or zeaxanthin (50 mg/kg) in the absence and presence of ezetimibe (100 mg/kg), respectively. B: Concentrations of β-carotene in *Bco1*^*−/−*^ mice treated and not treated with ezetimibe. C: Concentrations of oxidized zeaxanthin in *Bco2*^*−/−*^ mice treated and not treated with ezetimibe. WAT, white adipose tissue.

To determine the contribution of NPC1L1 to the absorption of carotenoids, we took advantage of *Bco1*^*−/−*^ and *Bco2*^*−/−*^ mice. These mice allow determining β-carotene and zeaxanthin carotenoid uptake levels, respectively, because they cannot metabolize these carotenoids (18, 32). Thus, we fed *Bco1*^*−/−*^ and *Bco2*^*−/−*^ mice with diets supplemented with 25 mg/kg of β-carotene or 50 mg/kg zeaxanthin in the presence or absence of ezetimibe, a known NPC1L1 inhibitor (33) (Fig. 1A). After two weeks of dietary intervention, we sacrificed the mice and determined the concentration of carotenoids across select tissues by quantitative HPLC analysis. *Bco1*^*−/−*^ mice absorbed significant amounts of β-carotene and displayed serum concentrations of 4 μM after the short intervention period (Fig. 1B). β-Carotene became also detectable in the liver of these mice. We observed no significant differences in β-carotene concentrations between treated and untreated animals, suggesting that ezetimibe had no effect on the absorption of this pure hydrocarbon under the applied conditions (Fig. 1B).

We next analyzed *Bco2*^*−/−*^ mice supplemented with zeaxanthin in the presence and absence of ezetimibe. Consistent with prior studies, zeaxanthin existed mainly as oxidized metabolite in tissues of *Bco2*^*−/−*^ mice (19). Untreated mice absorbed significantly more zeaxanthin than treated mice, as reflected in higher serum and liver concentrations of the carotenoid (Fig. 1C). The adrenal glands, expressing high levels of SR-B1 and Aster-B (25), exhibited the highest concentration of oxidized zeaxanthin in control mice. Importantly, ezetimibe treatment significantly reduced zeaxanthin accumulation in the adrenal glands, underscoring NPC1L1’s contribution to zeaxanthin absorption. A similar trend was observed in visceral adipose tissue, though the difference between treatment groups did not reach statistical significance (Fig. 1C).

### Characterization of the lipid-binding domain of Aster-C

**Fig. 2:**
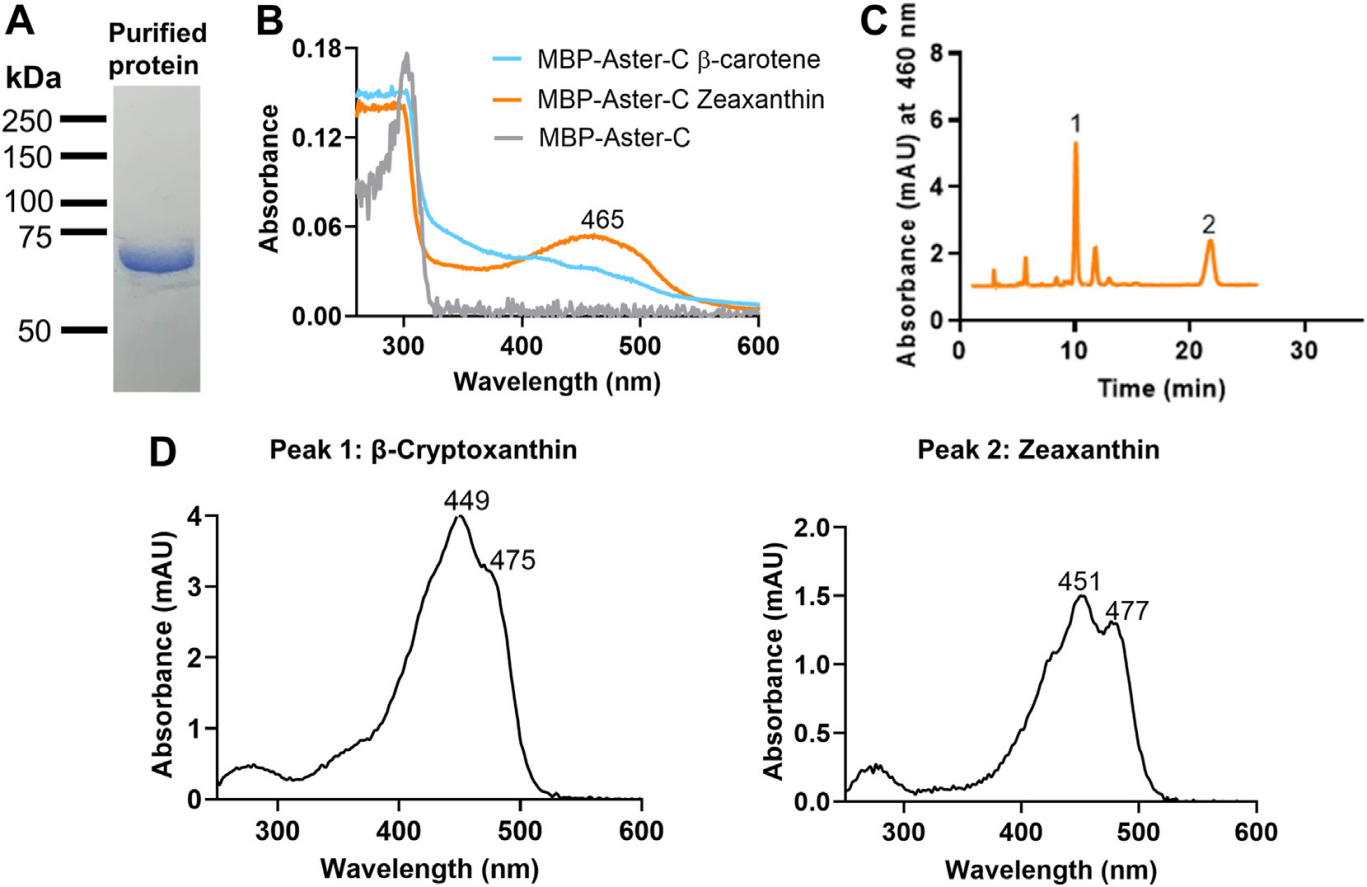
The StARD-like lipid binding domain of Aster-C binds carotenoids. A: SDS-PAGE for purified MBP-Aster-C protein. B: UV-visible absorption spectra of the MBP-Aster-C carotenoprotein complexes isolated from β-carotene and zeaxanthin-producing bacteria and apo-protein spectrum. C: HPLC chromatogram of carotenoids extracted from the carotenoprotein complex of MBP-Aster-C expressed in zeaxanthin-producing bacteria. D: UV-visible spectra of peaks 1 (β-cryptoxanthin) and 2 (zeaxanthin).

*Gramd1c*, encoding Aster-C, is highly expressed in the gastrointestinal tract (23, 24), though its capacity to bind carotenoids remains unstudied. To explore this, we expressed the StART domain of Aster-C (amino acids 100–312) as a maltose-binding protein (MBP) fusion in *E. coli* engineered to produce β-carotene and zeaxanthin. To test whether the recombinant MBP-Aster-C protein bound these carotenoids, we conducted a spectroscopic analysis with the affinity-purified carotenoprotein complexes (Fig. 2A). The MBP-Aster-C expressed in zeaxanthin-producing bacteria showed two absorption peaks: one in the UV and another in the visible range (Fig. 2B). The UV peak for MBP-Aster-C from *E. coli* producing β-carotene was comparable but the spectrum did lack a maximum absorption peak in the visible range, suggesting that no specific carotenoprotein complex was formed (Fig. 2B). The unique fine structure of the UV/visible spectra of the purified carotenoprotein complex from zeaxanthin producing bacteria, as compared to unbound carotenoids, indicated specific interactions between the carotenoid chromophore and Aster-C protein (Fig. 2). This interaction shifted the absorption maximum from 450 nm (unbound zeaxanthin) to 465 nm in the carotenoprotein complex (Fig. 2B and D).

To further confirm carotenoid binding to Aster-C, we denatured the purified carotenoprotein complex and extracted the bound carotenoids for HPLC analysis (Fig. 2C and D). Lipid extracts from MBP-Aster-C produced in zeaxanthin-synthesizing *E. coli* revealed peaks with the retention time and spectral characteristics of β-cryptoxanthin and zeaxanthin (Fig. 2C and D). Notably, no β-carotene was detected though it existed in the bacterial strain as precursor of β-cryptoxanthin and zeaxanthin. These results indicated that Aster-C forms carotenoprotein complexes with β-cryptoxanthin and zeaxanthin.

### Effects on Aster-C knockdown for vitamin A metabolism

**Fig. 3:**
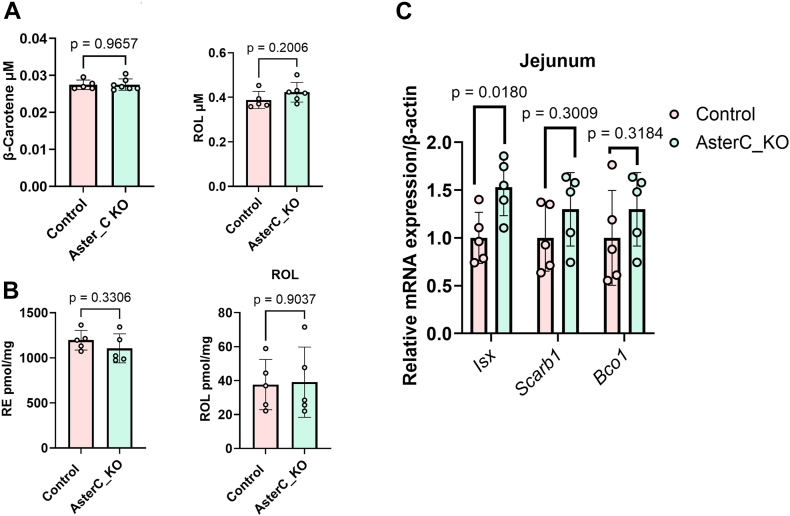
β-Carotene metabolism in *Gramd1c*^*−/−*^ mice. A: Serum concentrations of β-carotene and all-*trans*-retinol (ROL) in *Gramd1c*^*−/−*^ (Aster_KO). B: Hepatic concentrations of retinyl ester (RE) and ROL in *Gramd1c*^*−/−*^ (Aster_KO) and wild type control mice. C: qRT PCR analysis of mRNA levels of Intestine specific homeodomain (*Isx*), SR-B1 (encoded by *Scarb1*), and *Bco1* genes.

Enterocytes are the major site for retinoid biosynthesis in mice (16, 19, 32). Native BCO1 has been described as a soluble protein of intestinal enterocytes (34, 35, 36). Therefore, it is not clear how it acquires its lipophilic substrates. To study whether Aster proteins facilitate this interaction, we took advantage of a recently established Aster-C knockout mouse (29). Thus, we fed Aster-C-deficient and control mice a diet supplemented with β-carotene (25 mg/kg) for two weeks. Following the dietary intervention, we measured β-carotene and ROL in serum and ROL and RE in liver. The analysis revealed that the concentration of β-carotene and its retinoid metabolites were comparable between genotypes (Fig. 3A and B). qRT-PCR analysis revealed that jejunal mRNA levels of *Isx* (encoding intestine specific homeodomain transcription factor), *Scarb1*, and *Bco1* were also comparable between genotypes (Fig. 3C). These results implied that Aster-C deficiency did not interfere with β-carotene conversion to retinoids or altered the mRNA levels of genes encoding the involved proteins.

### *Gramd1* gene expression is independent of ISX

**Fig. 4:**
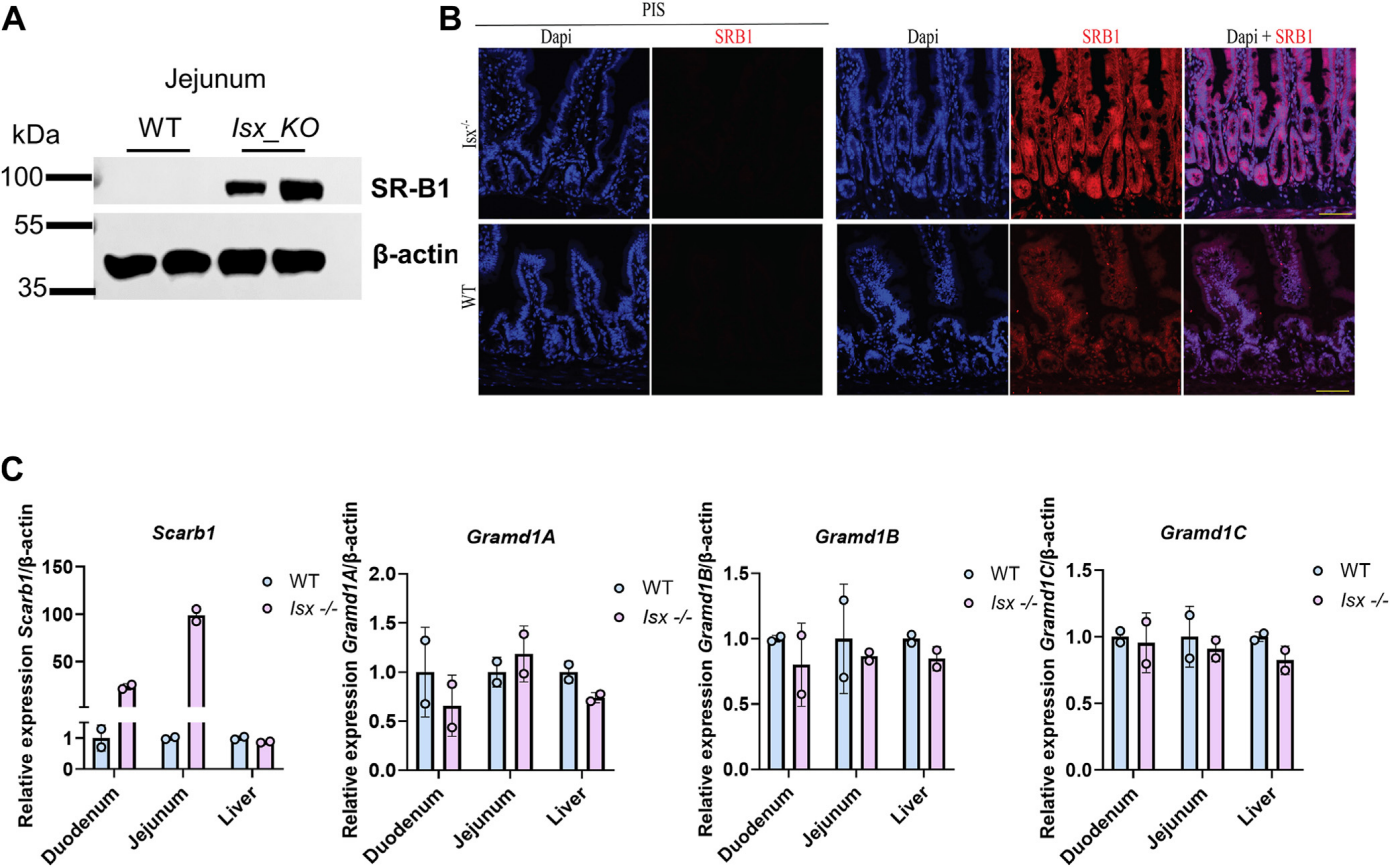
*Gramd1*a, *b*, and *c* genes are not regulated by ISX in the mouse intestine. A: Western blot for SR-B1 protein in control (WT) and *Isx*^*−/−*^ mice. Fifty microgram jejunal protein extracts were loaded per lane. β-Actin was used as loading control. B: Immunostaining for SR-B1 in jejunal sections of control (WT) and *Isx*^*−/−*^ mice. PIS, serum control, DAPI (blue), and SR-B1 (red). C: qRT-PCR analysis of the expression of *Scarb1* and *Gramd1a*, *b*, and *c* genes in jejunal RNA preparations of control (WT) and *Isx*^*−/−*^ mice.

ISX is a major regulator of vitamin A production in enterocytes (14). ISX controls the expression of *Scarb1* and *Bco1*, genes encoding key proteins for carotenoid uptake and conversion to vitamin A while its own expression is modulated by dietary vitamin A through retinoid signaling (37, 38, 39). Therefore, we assumed that an Aster protein acting downstream of SR-B1 would display a similar regulation of its expression pattern. To clarify this, we analyzed *Scarb1* expression and compared it to the expression of *Gramd1* genes in wild type and ISX-deficient mice. Western blot analysis showed that SR-B1 protein levels were higher in ISX-deficient than in wild type mice (Fig. 4A). This increase in SR-B1 was confirmed by immunohistochemistry, which demonstrated strong staining for SR-B1 in the jejunal villi of ISX-deficient mice, whereas significant less staining was observed in wild type mice (Fig. 4B). Consistently, *Scarb1* mRNA levels were increased over 30-fold in the duodenum and nearly 100-fold in the jejunum of *Isx*^*−/−*^ mice compared to wild-type controls (Fig. 4C). In contrast, mRNA levels for all three *Gramd1* genes encoding Aster proteins were comparable in ISX-deficient and wild type, indicating that ISX does not regulate these genes in intestinal enterocytes. Notably, ISX deficiency did not affect hepatic *Scarb1* mRNA expression, indicating that ISX selectively modulates the vitamin A synthesis pathway in the intestine without affecting SR-B1 hepatic functions, emphasizing SR-B1’s role in balancing both intestinal carotenoid and hepatic cholesterol metabolism.

### BCO1 displays a conserved amphipathic helix for membrane interaction

**Fig. 5:**
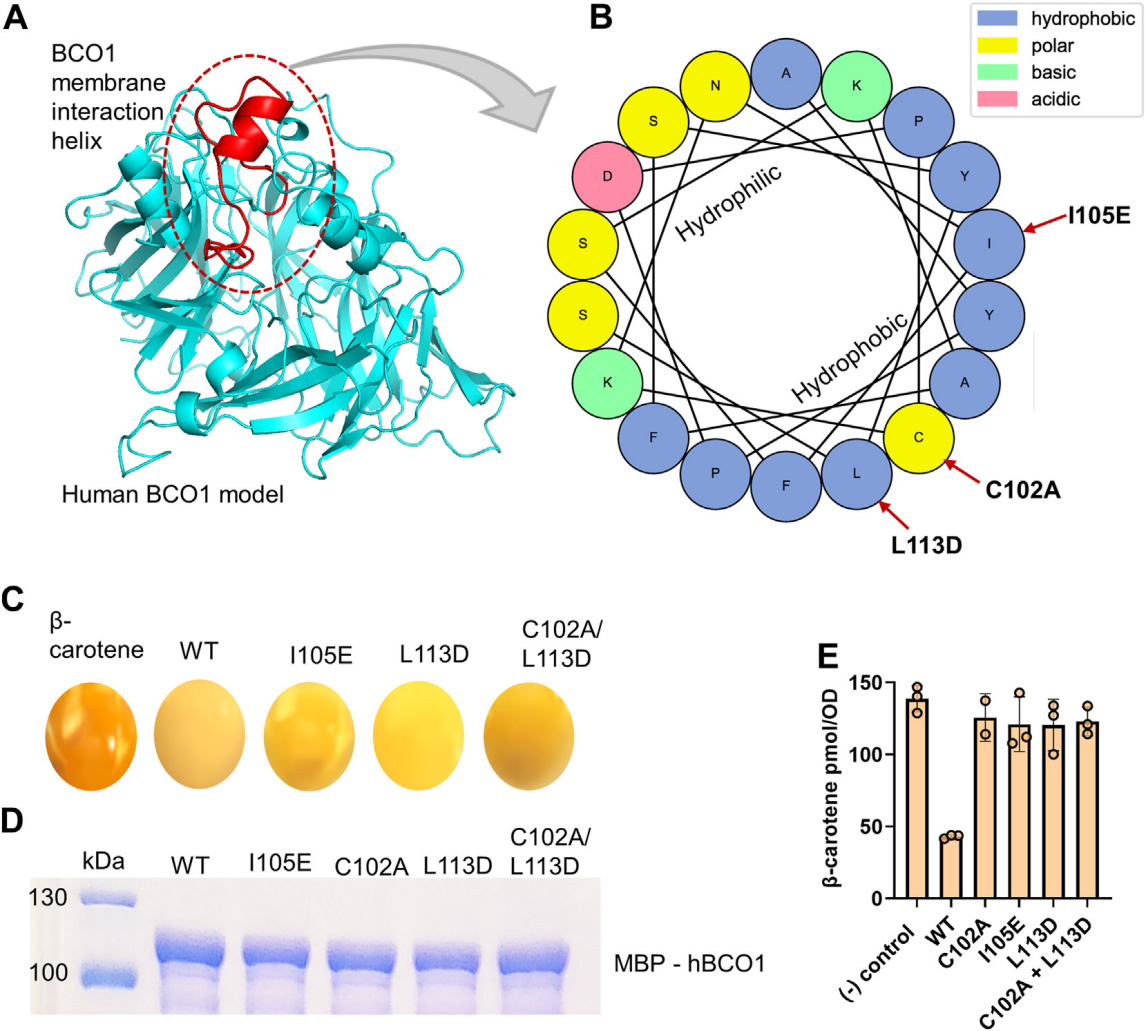
Membrane interaction of β-carotene-oxygenase 1. A: Structural model for human BCO1. The putative membrane interaction helix is highlighted in red. B: Helical wheel plot for the membrane interaction helix of BCO1. The mutated amino acids are indicated with arrows. C: Color comparison. of β-carotene-synthesizing *E. coli* strains expressing different human BCO1 variants. D: SDS-PAGE for the purified human BCO1 mutant variants. E: β-carotene content of β-carotene-synthesizing *E. coli* strains expressing different human BCO1 variants.

An alternative to the delivery of β-carotene by Aster proteins would be a direct interaction of BCO1 with plasma membrane. Recent structural analyses of a related insect carotenoid cleavage enzyme, NinaB, revealed a surface region near the entrance to the substrate tunnel that facilitates membrane interaction (40). This region starts with a “PDPC(+)” motif, (+) indicating a cationic residue, that is conserved in BCO1 (Fig. 5A and B). In the NinaB structure, these amino acids form an amphipathic α-helix that is critical for enzymatic activity (40). To study whether this helix motif is involved in the acquisition of the substrate, we performed site-directed mutagenesis with BCO1 to disrupt the amphipathic α-helix. For this purpose, we converted two hydrophobic side residues into polar side residues (Fig. 5B). Additionally, we mutated the cysteine of the PDPC + motif into alanine. We then expressed the mutant BCO1 variants in *E. coli* capable of synthesizing β-carotene to determine their enzymatic activity. The outcome of this experiment indicated that wild type BCO1 converted β-carotene to retinoids as seen by the color shift of the bacteria pellet from yellow to pale and the reduced β-carotene content of the bacteria. In contrast, changes of amino acids in the proposed membrane interaction region significantly reduced enzymatic activity of BCO1 as seen by the colors and high β-carotene content of the bacteria (Fig. 5C and E). SDS-PAGE analyses revealed that alterations in enzymatic activity were unrelated to protein expression or solubility changes of the generated BCO1 mutant variants (Fig. 5D). Enzyme assays with β-carotene solubilized in detergent micelles revealed that human BCO1 converted β-carotene into retinaldehyde, whereas all mutant variants showed reduced retinaldehyde production (supplemental Fig. S1). Thus, we hypothesize that human BCO1 interacts with membranes to extract its lipophilic substrate as described for insect NinaB (40).

### Effects of Aster-C on xanthophyll absorption and distribution

**Fig. 6:**
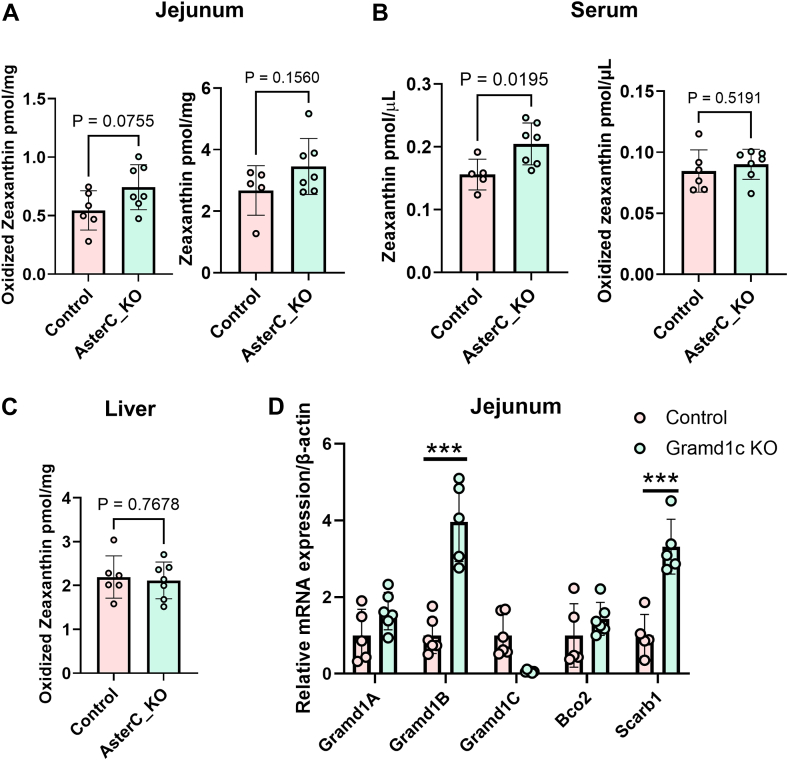
*Gramd1c* knockout mice display altered zeaxanthin absorption. A–C: Concentration of parent zeaxanthin and oxidized zeaxanthin in the jejunum, serum, and liver of control and *Gramd1c*^*−/−*^ (AsterC_KO) mice. D: qRT PCR analysis of the mRNA expression of *Gramd1* genes, *Bco2*, and *Scarb1* in the jejunum of control and *Gramd1c*^*−/−*^ (AsterC_KO) mice.

To study the effect of Aster-C deficiency on zeaxanthin absorption and metabolism, we fed *Gramd1c*^*−/−*^ and control mice a diet containing 250 mg/kg zeaxanthin. We choose this relatively high amount of the carotenoid because *Gramd1c*^*−/−*^ and wild type control mice express BCO2, which catabolizes absorbed zeaxanthin (19). After two weeks of feeding, we analyzed zeaxanthin across various organs of the animals. Zeaxanthin presented as both the parent compound and in oxidized form in mouse tissues of both genotypes (Fig. 6) (18, 41). In the jejunum, knockout mice showed a trend toward higher zeaxanthin concentrations though the values did not reach statistical significance (Fig. 6A). Notably, Aster-C deficient mice exhibited significantly higher serum levels of parent zeaxanthin compared to wild-type mice, suggesting that more zeaxanthin was absorbed in the absence of Aster-C. No differences were observed for liver where zeaxanthin existed mainly in oxidized form (Fig. 6C). Analysis of the expression of *Gramd1a* and *b* mRNA by qRT-PCR analysis revealed increased expression in the jejunum of *Gramd1c*^*−/−*^ mice when compared to wild type mice (Fig. 6D). Additionally, there was a significant increase in the expression of *Scarb1* gene in RNA preparation from the intestine of *Gramd1c*^*−/−*^ mice when compared to wild type mice (Fig. 6D). These outcomes suggested that Aster-C deficiency does not disrupt zeaxanthin metabolism, as Aster-B may compensate for the loss of function. However, elevated serum zeaxanthin in *Gramd1c*^*−/−*^ mice indicated that in the absence of Aster-C more zeaxanthin was absorbed or escaped mitochondrial catabolism by BCO2.

## Discussion

**Fig. 7:**
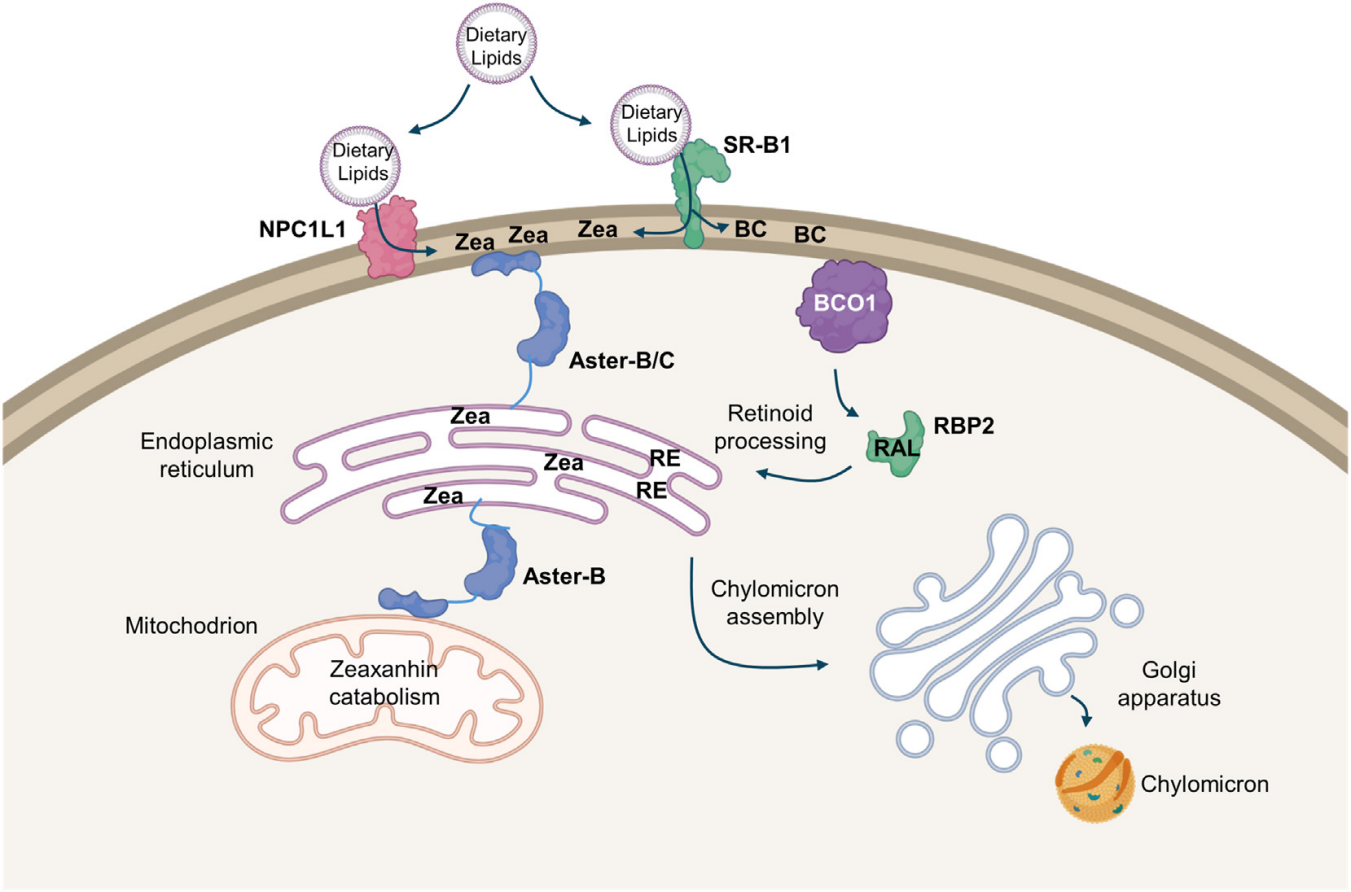
Scheme of carotenoid metabolism of intestinal enterocytes. Carotenoids along with other dietary lipids are solubilized in mixed micelles. β-Carotene (BC) absorption is facilitated by SR-B1, which loads it into plasma membrane. BCO1 converts BC to retinaldehyde (RAL). Cellular retinoid-binding protein 2 (RBP2) facilitates RAL transport and metabolism to retinyl esters (RE). Zeaxanthin (Zea) absorption is facilitated by both SR-B1 and NPC1L1 which load Zea into the plasma membrane. Aster-B and C facilitate the transport of Zea to the endoplasmic reticulum. Here, Zea faces two fates: packaging into chylomicrons alongside other lipids and Aster-B-dependent transport to mitochondria and catabolism by BCO2.

Carotenoids are essential nutrients with a well-recognized impact on ocular health (4). Their absorption and metabolism in the intestine involve a complex interplay between multiple transporters and enzymes (14). Genetic polymorphism in genes encoding these proteins is associated with serum and ocular carotenoid concentrations (2). Although carotenoid uptake via SR-B1 and NPC1L1 has been previously studied in mice (13, 14), the specific contribution of the transporters and the mechanisms guiding carotenoids to their metabolizing enzymes remain incompletely understood. Here, we provide evidence that β-carotene and zeaxanthin engage different proteins for their trafficking and metabolism in enterocytes (for the current model see Fig. 7).

Previous studies addressing ezetimibe’s effects on carotenoid absorption were conducted in human Caco2 cells and reported that both xanthophyll and carotene uptake was affected by the drug (42). The generation of knockout mice for BCO1 and BCO2 established murine models that similar to humans absorb significant amounts of carotenoids intact and distribute them in the body. Using these mouse models, we showed that NPC1L1 plays a more selective role in the absorption of carotenoids, as evidenced by the differential effects of ezetimibe, a known NPC1L1 inhibitor. In BCO2 knockout mice, ezetimibe treatment significantly reduced zeaxanthin absorption, while it had no impact on β-carotene levels in BCO1-deficient mice. Accordingly, the drug did not change the expression of retinoid-responsive genes in the intestine. This finding suggests that β-carotene absorption occurs NPC1L1-independently mainly by SR-B1, which is subject to regulation by retinoid signaling in the intestine (39).

Our experiments revealed that zeaxanthin relies more heavily on NPC1L1-mediated transport than β-carotene as its levels decreased in *Bco2*^*−/−*^ mice in the presence of the drug. The selective interaction between NPC1L1 and zeaxanthin, potentially driven by structural features like the presence of hydroxyl groups in the carotenoid, underscores a specific role of NPC1L1 in carotenoid metabolism. Supporting this, previous studies have shown that the absorption of lycopene, another pure hydrocarbon carotenoid, was not affected by ezetimibe in mice but increased with the overexpression of SR-B1 in the intestine (43). Additionally, SNPs in NPC1L1 are linked to variations in macular pigment optical density in clinical studies, aligning with a role of NPC1L1 role in zeaxanthin metabolism (11, 44).

Therefore, our findings implicate potential interactions between lipid-lowering drugs like ezetimibe and zeaxanthin metabolism. While ezetimibe effectively reduces cholesterol absorption by targeting specific pathways, primarily to lower LDL cholesterol levels and reduce cardiovascular disease risk (45); its impact on zeaxanthin uptake may be considered as an adverse side effect. Therefore, ongoing research into novel inhibitors of cholesterol uptake (23, 46), should aim to optimize cholesterol management while potentially mitigating unintended effects on carotenoid absorption.

Our studies further explored how carotenoids interact with the BCO1 and BCO2 enzymes in enterocytes. We demonstrated that the StART domain of Aster-C can bind zeaxanthin, indicating a potential role in carotenoid transport within enterocytes. Accordingly, experiments in Aster-C-deficient mice revealed that provitamin A metabolism remains intact, as shown by stable serum retinol and hepatic retinoid levels. This suggests that β-carotene uptake and conversion to vitamin A are Aster-independent and sustained through a direct interaction between BCO1 and PM. This interaction likely involves an amphipathic α-helix near BCO1’s substrate tunnel, which facilitates direct access of membrane-bound β-carotene to the enzyme’s catalytic site. Site-directed mutagenesis and enzyme assays in *E. coli* confirmed the critical role of this α-helix, with mutations significantly impairing enzymatic turnover. This membrane interaction mechanism parallels the function of NinaB in insects (40).

Our findings highlight BCO1's specialized adaptations for efficient provitamin A metabolism, distinct from BCO2's role in processing non-provitamin A carotenoids in mitochondria. However, it remains to be investigated whether Aster proteins are required to absorb β-carotene intact and incorporate it into chylomicrons. Moreover, Aster proteins role in β-cryptoxanthin metabolism needs to be investigated because its conversion to retinoids involve both BCO1 and BCO2 (27).

Our conclusion of an Aster-independent retinoid production from β-carotene is further supported by the observation that *Gramd1* gene expression is not regulated by ISX, the transcription factor that controls key elements in vitamin A production, such as SR-B1 and BCO1 (38). Both genes are highly expressed in enterocytes of vitamin A-deficient mice (37). In vitamin A sufficiency, retinoid signaling induces ISX expression that represses *Scarb1* and *Bco1* gene expression in the intestine (47, 48). The absence of ISX regulation underscores a functional distinction between Aster proteins and the machinery dedicated to vitamin A homeostasis.

Previously, we observed that Aster proteins specifically facilitate the intracellular transport of zeaxanthin and lutein, from the plasma membrane to mitochondria, whereas β-carotene does not follow this transport route (26). This selective movement of xanthophyll by Aster proteins highlights their specialized role in the metabolism of non-provitamin A carotenoids, reinforcing that Aster proteins are pivotal to xanthophyll handling rather than vitamin A synthesis and storage. Notably, Aster-C deficiency led to an increased bioavailability of zeaxanthin. The observed upregulation of *Gramd1b*, encoding Aster-B, in Aster-C-deficient mice, suggests a compensatory response, where increased Aster-B enhances zeaxanthin uptake and transport within enterocytes. This compensatory mechanism points to functional redundancy within the Aster family and has already been observed for cholesterol transport by Asters in this mouse model (29).

In conclusion, our studies reveal distinct mechanisms governing β-carotene and zeaxanthin absorption and metabolism in enterocytes (Fig. 7), highlighting the specialized roles of NPC1L1 and Aster proteins in these processes. We demonstrated that NPC1L1 preferentially facilitated zeaxanthin uptake, which can be disrupted by ezetimibe, whereas β-carotene appears to rely on the SR-B1 pathway for intestinal absorption. Additionally, our findings on Aster-C suggest it plays a role in intracellular xanthophyll trafficking. In contrast, the direct interaction between BCO1 and the plasma membrane ensures that β-carotene can be efficiently converted to vitamin A independently of Aster proteins. Together, these insights deepen our understanding of the molecular pathways that regulate carotenoid bioavailability and metabolism, with implications for optimizing dietary interventions and therapeutic strategies targeting nutrient absorption and cholesterol management.

## Data availability

The authors confirm that the data supporting the findings of this study are contained within the article and the supplementary information. The raw data are available upon request from the corresponding author.

## Supplemental data

This article contains supplemental data.

## Conflict of interest

The authors declare that they have no conflicts of interest with the contents of this article.
