# Supplementary material for: Distinct pathways for the absorption and metabolism of β-carotene and zeaxanthin in the mouse intestine
Source: J Lipid Res. 2025 Feb 17;66(3):100758. doi: 10.1016/j.jlr.2025.100758 (PMC11957524; doi:10.1016/j.jlr.2025.100758)
Supplement: Supplemental data [file mmc1.docx]

**Supporting Information**

**Distinct pathways for the absorption and metabolism of β-carotene and zeaxanthin in the mouse**

**Intestine**

**
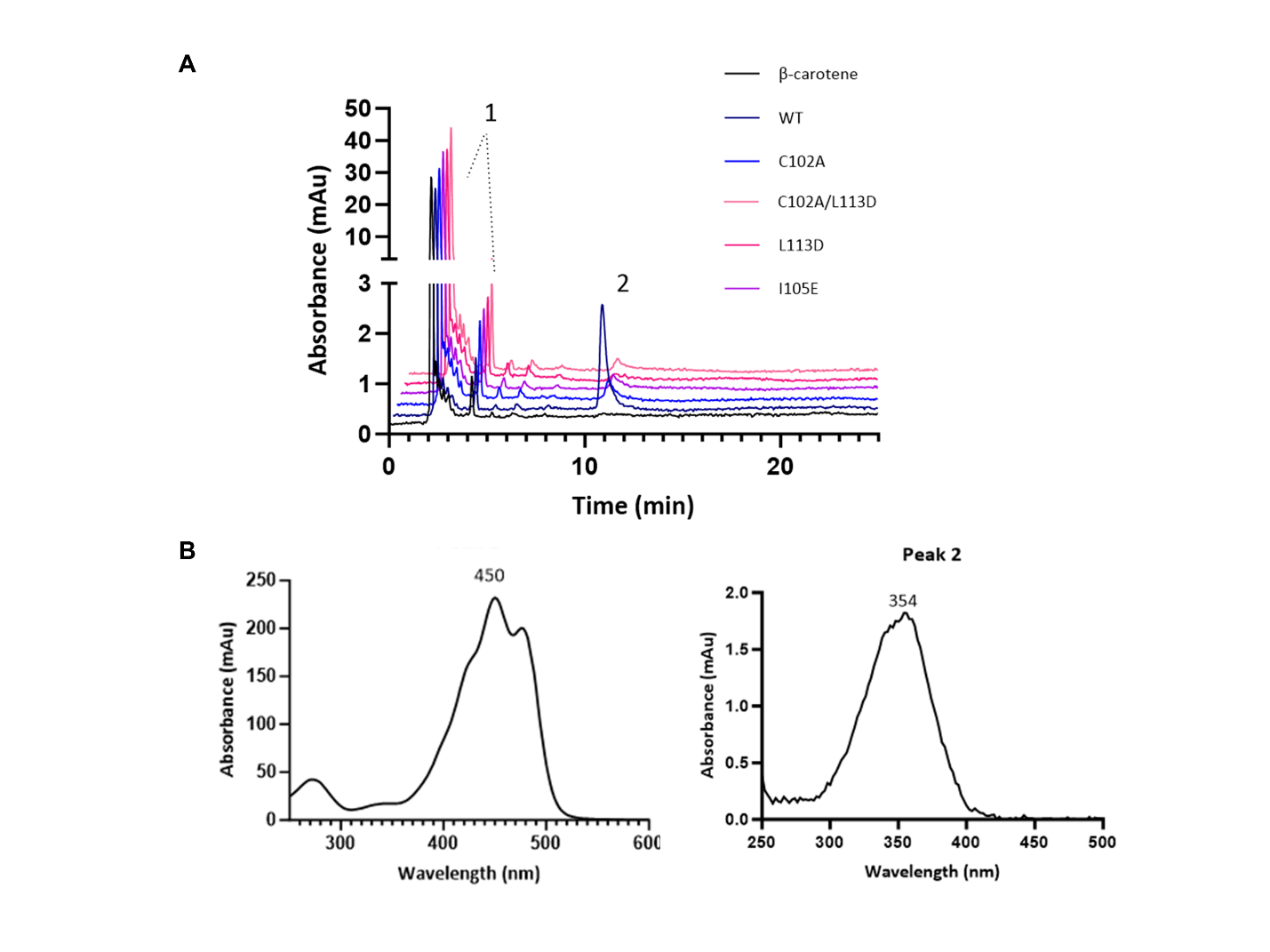
**

**Figure S1. Catalytic activity of different purified human BCO1 variants. A.** HPLC chromatograms of lipid extracts of enzyme assays with different human BCO1 variants. β-carotene- substrate (black), WT (dark-blue), C102A (blue), C102A/L113D (pink), L113D (dark pink) and I105E (purple). **B.** UV-Visible absorption spectra of β-carotene (peak 1) and all-*trans*-retinal-oxime (anti) (peak 2).
